# The bystander effect contributes to the accumulation of senescent cells in vivo

**DOI:** 10.1111/acel.12848

**Published:** 2018-11-21

**Authors:** Paulo F. L. da Silva, Mikolaj Ogrodnik, Olena Kucheryavenko, Julien Glibert, Satomi Miwa, Kerry Cameron, Abbas Ishaq, Gabriele Saretzki, Sushma Nagaraja‐Grellscheid, Glyn Nelson, Thomas von Zglinicki

**Affiliations:** ^1^ The ABC – Newcastle University Ageing Biology Centre Institute for Cell and Molecular Biology Campus for Ageing and Vitality Newcastle University Newcastle upon Tyne UK; ^2^ Department of Biosciences Durham University Durham UK; ^3^ Arts and Sciences Faculty, Molecular Biology and Genetics Near East University Mersin Turkey; ^4^ Present address: Institute for Genome Stability in Ageing and Disease Cologne Excellence Cluster for Cellular Stress Responses in Aging‐Associated Diseases (CECAD) University of Cologne Joseph‐Stelzmann‐Str. 26 Cologne 50931 Germany; ^5^ Present address: Federal Institute for Risk Assessment Max‐Dohrn‐Str. 8‐10 Berlin 10589 Germany; ^6^ Present address: Computational Biology Unit Department of Biosciences University of Bergen Bergen 5006 Norway

**Keywords:** aging, bystander, muscle, senescence, skin

## Abstract

Senescent cells accumulate with age in multiple tissues and may cause age‐associated disease and functional decline. In vitro, senescent cells induce senescence in bystander cells. To see how important this bystander effect may be for accumulation of senescent cells in vivo, we xenotransplanted senescent cells into skeletal muscle and skin of immunocompromised NSG mice. 3 weeks after the last transplantation, mouse dermal fibroblasts and myofibres displayed multiple senescence markers in the vicinity of transplanted senescent cells, but not where non‐senescent or no cells were injected. Adjacent to injected senescent cells, the magnitude of the bystander effect was similar to the increase in senescence markers in myofibres between 8 and 32 months of age. The age‐associated increase of senescence markers in muscle correlated with fibre thinning, a widely used marker of muscle aging and sarcopenia. Senescent cell transplantation resulted in borderline induction of centrally nucleated fibres and no significant thinning, suggesting that myofibre aging might be a delayed consequence of senescence‐like signalling. To assess the relative importance of the bystander effect versus cell‐autonomous senescence, we compared senescent hepatocyte frequencies in livers of wild‐type and NSG mice under ad libitum and dietary restricted feeding. This enabled us to approximate cell‐autonomous and bystander‐driven senescent cell accumulation as well as the impact of immunosurveillance separately. The results suggest a significant impact of the bystander effect for accumulation of senescent hepatocytes in liver and indicate that senostatic interventions like dietary restriction may act as senolytics in immunocompetent animals.

## 1 INTRODUCTION

Senescent cells accumulate in many tissues during aging. They produce ROS and are inferior in terms of mitochondrial function and metabolism (senescence‐associated mitochondrial dysfunction SAMD) (Korolchuk, Miwa, Carroll & von Zglinicki, 2017), limit tissue regeneration (Jurk et al., 2014) and secrete a host of bioactive molecules, specifically pro‐inflammatory cytokines, chemokines and matrix‐remodelling enzymes (senescence‐associated secretory phenotype SASP) (Coppe et al., 2008), thereby contributing to age‐related tissue dysfunction. Accordingly, genetic or drug‐mediated specific ablation of senescent cells ameliorates a wide range of age‐associated disabilities and diseases in mice (Baar et al., 2017; Baker et al., 2011, 2016).

Cell senescence can be triggered by replicative exhaustion or stressors, specifically oncogenic and DNA‐damaging stress. Moreover, pre‐existing senescent cells in vitro are capable of inducing a senescent phenotype in surrounding bystander cells via integrated ROS‐ and NF‐κB‐dependent signalling pathways (Acosta et al., 2013; Nelson et al., 2012). It has been suggested that this senescence‐induced bystander senescence might be a relevant trigger of senescent cell accumulation in vivo, based on focal clustering of senescent cells in old mouse livers (Nelson et al., 2012) and of SASP‐mediated accumulation of senescent cells around pre‐neoplastic lesions (Acosta et al., 2013). In accordance, autologous transplantation of senescent fibroblasts into healthy knee joints resulted in the development of an osteoarthritis‐like condition in mice (Xu et al., 2017). Very recently, it was shown that intraperitoneal transplantation of relatively low numbers (0.5–1 × 10^6^) of senescent cells caused persistent physical dysfunction in mice (Xu et al., 2018), indicating that senescent cells can induce a deleterious bystander effect in vivo. However, direct evidence that transplanted or pre‐existing senescent cells do induce senescence in surrounding tissues is still weak.

The impact of cell senescence for aging of skeletal muscle and the dermal layer of the skin has been questioned because the major cell types are slowly dividing (dermal fibroblasts) or not dividing at all (myofibres). However, the DNA damage response (DDR) induces a senescence‐like phenotype in postmitotic cells like neurons (Fielder, von Zglinicki & Jurk, 2017; Jurk et al., 2012) or retinal cells (Oubaha et al., 2016) characterized by production of senescence‐associated β‐galactosidase, SAMD and SASP. In the dermis, accumulation of fibroblasts with telomere dysfunction and other senescence markers has been observed in different mammalian species (Herbig, Ferreira, Condel, Carey & Sedivy, 2006; Wang et al., 2009). In mouse skeletal (gastrocnemius) muscle, expression of various senescence markers increased with age and decreased after selective ablation of p16‐expressing presumably senescent cells (Baker et al., 2016); however, the responsible cell type had not been identified.

After observing increased frequencies of multiple senescence markers in aging myofibres, we xenotransplanted small numbers of senescent human fibroblasts into mouse skeletal muscle and skin. Bioluminescent and fluorescent labelling enabled tracking of the injected cells in vivo for at least 3 weeks as well as their identification in cryosections in situ. We found that mouse cells surrounding the injection sites showed increased frequencies of multiple senescence markers when senescent cells (but not non‐senescent cells) were xenotransplanted. Comparing senescent cell accumulation rates in normal and immunocompromised mice under either ad libitum feeding or dietary restriction enabled separate estimations of bystander‐dependent versus cell‐autonomous senescent cell accumulation, indicating a significant and possibly major contribution of the bystander effect.

## 2 RESULTS

### 2.1 Myofibres in aging skeletal muscle show multiple markers of cell senescence

**Figure 1:**
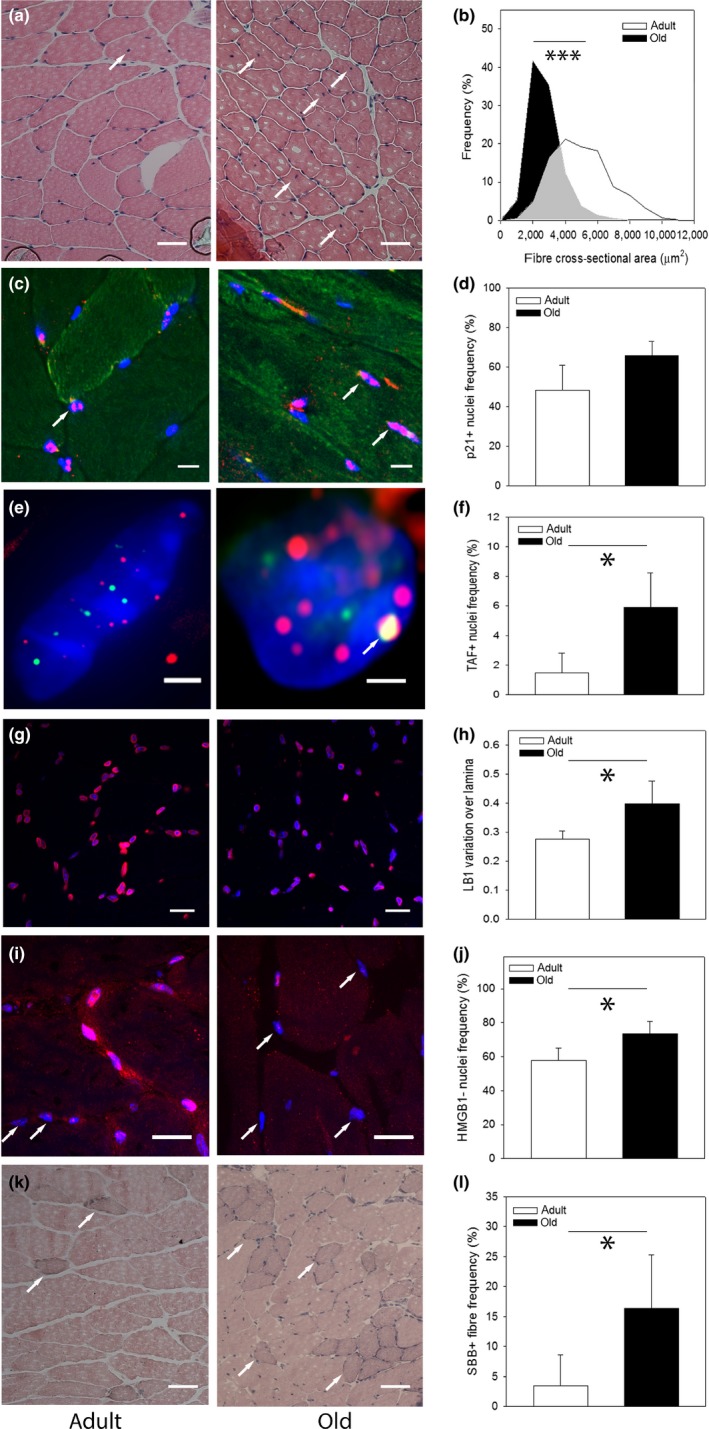
A senescent phenotype in aged myofibres. (a) Representative images of H&E‐stained cryosections of gastrocnemius muscle from C57Bl6 mice at 8 (left) and 32 (right) months of age. Scale bar 50 μm. Arrows indicate centrally nucleated fibres (CNF). (b) Frequency distributions of myofibre cross‐sectional areas in adult and old mice. Averages are different with *p* < 0.001 (Mann–Whitney *U* test). (c) Representative p21 immunofluorescence images. Red: p21, blue: DAPI, green autofluorescence. Arrows indicate examples of positive nuclei. Bar equals 15 μm. (d) Frequencies of p21‐positive nuclei in adult and old muscles. (e) Immuno‐FISH staining for telomeres (red) and γH2AX (green). Signal co‐localization (TAF) is marked by an arrow. Scale bar 2 μm. (f) Frequencies of TAF‐positive nuclei. (g) lamin B1 immunofluorescence. Red: lamin B1, blue: DAPI. Bar equals 20 μm. (h) Normalized pixel‐to‐pixel variation of laminar LB1 fluorescence intensity. (i) HMGB1 immunofluorescence (red). Blue: DAPI. Arrows indicate examples of HMBG1‐negative nuclei. Scale bar 20 μm. (j) Frequencies of HMGB1‐positive nuclei. (k) SBB plus Nuclear Fast Red‐stained cryosections. SBB‐positive fibres appear dark (examples indicated by arrows). Scale bar 50 μm. (l) Frequencies of SBB‐positive fibres. All data are mean ± *SD*, three animals/group. *p < 0.05

Fibre thinning is a frequently used marker of muscle aging and sarcopenia (Miljkovic, Lim, Miljkovic & Frontera, 2015). Centrally nucleated fibres (CNF) are regarded as a sign of fibre regeneration following age‐associated degeneration (Sayed et al., 2016). Comparing adult (8 months) and old (32 months) mice (Figure 1a), we found decreased fibre cross‐sectional area (CSA, Figure 1b) and increased frequencies of myofibres with centrally located nuclei (CNF, Figure 1a, Supporting information Figure S1A) in old muscle.

We next measured multiple markers of cell senescence at single nucleus/myofibre level in the same muscle samples. p21 (CIP1) is a major mediator of the senescent cell cycle arrest and the SAMD (Choudhury et al., 2007; Passos et al., 2010). Frequencies of p21 (CIP1)‐positive nuclei tended to increase in muscles from old mice (Figure 1c, d). While p21 expression is not specific to cell senescence, telomere‐associated DNA damage foci (TAF) have been identified as more specific marker of cell senescence in mice and humans (Hewitt et al., 2012; Jurk et al., 2014). Nuclei containing TAF were more frequent in old myofibres (Figure 1e, f). Loss and nuclear redistribution of lamin B1, and specifically a more heterogeneous distribution along the nuclear lamina, has been described as a cell senescence biomarker (Freund, Laberge, Demaria & Campisi, 2012). We found that lamin B1 fluorescence intensity tended to decrease in old muscles (Figure 1g, Supporting information Figure S1B). Moreover, the lamin B1 staining intensity of the nuclear lamina was more heterogeneous in old animals (Figure 1h). Loss of HMGB1 (alarmin) is another well‐established marker of senescence (Davalos et al., 2013), and we found more HMGB1‐negative myofibre nuclei in old skeletal muscle (Figure 1i, j). Finally, Sudan Black B (SBB), reacting with lipofuscin, has been established as a sensitive indicator of senescence‐associated β‐galactosidase activity and thus of cell senescence (Georgakopoulou et al., 2013). Frequencies of SBB‐positive fibres were higher in old muscles (Figure 1k, l).

Increased senescence in old muscles was confirmed by qPCR for p16 (but not p21) and SASP markers IL‐1α, IL‐1β, IL‐6 and TNF‐α (Supporting information Figure S1C) in agreement with earlier data (Baker et al., 2016). However, despite increased systemic inflammation in old mice (Jurk et al., 2014; Xu et al., 2015), we found no evidence for increased abundance of SASP cytokines at protein level in old muscle (Supporting information Figure S1D).

### 2.2 Senescence marker‐bearing myofibres are thinner

Together, these data show that aged myofibres in mice show multiple hallmarks of cell senescence, both nuclear and cytoplasmic. This suggests that in aging muscle, similar to other tissues containing largely slow‐ or non‐dividing cells, for example, brain (Jurk et al., 2012), retina (Oubaha et al., 2016), liver (Jurk et al., 2014; Ogrodnik et al., 2017; Wang et al., 2009) or dermis (Herbig et al., 2006; Wang et al., 2009), myofibres with a senescent phenotype accumulate.

**Figure 2:**
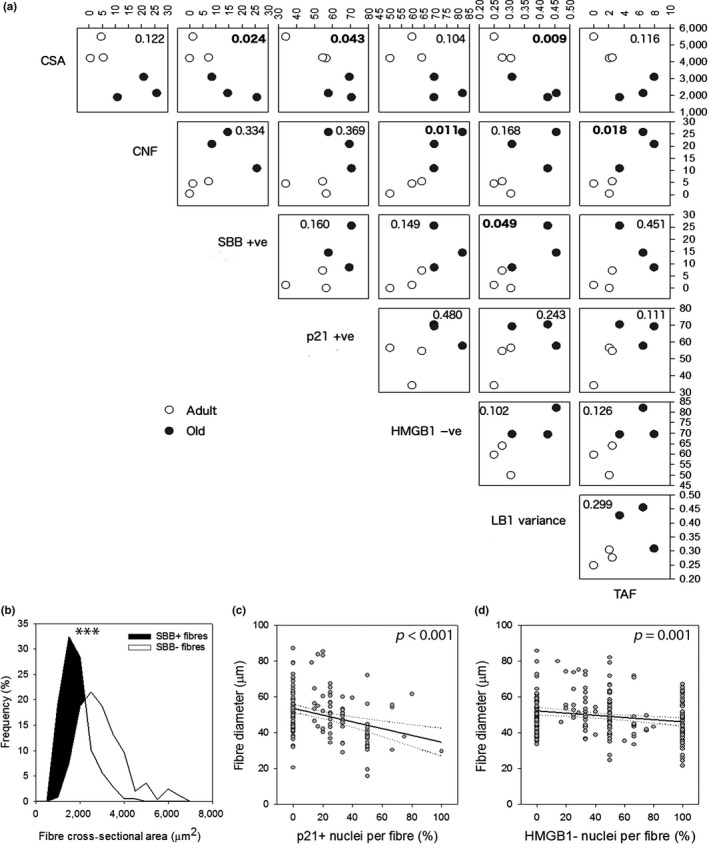
Nuclear and cytoplasmic markers of myofibre senescence are associated with fibre thinning. (a) Inter‐individual Pearson product moment correlations between muscle aging markers (cross‐sectional area CSA, frequency of centrally nucleated fibres CNF) and senescence markers (SBB + fibres, p21 + nuclei, HMGB1‐ nuclei, LB1 variance over nuclear lamina and TAF+ nuclei). *p* values are shown (*p* values below 0.05 in bold). Open circles: adult; filled circles: old animals. (b) Frequency distributions of cross‐sectional area of SBB‐positive and SBB‐negative myofibres in old muscles. *p* < 0.001 for difference between means (Mann–Whitney *U* test). (c) and (d) Correlations between frequencies of p21+ nuclei (c) or HMGB1‐ nuclei (d) per fibre cross‐section and minimum Feret diameter of the fibre. Regression line and 95% confidence interval (dotted) are shown, and *p* values for the correlations are indicated

To address potential functional consequences of myofibre senescence, we first assessed the correlations between markers of fibre aging and senescence at the level of individual mice (Figure 2a). Myofibre cross‐sectional area was associated with frequencies of SBB+ fibres, p21+ nuclei and with lamin B1 variance, while CNF frequencies were associated with HMGB1‐ and TAF+ nuclei frequencies (Figure 2a). At single fibre level, fibre thinning was independent from CNF presence: The average fibre cross‐sectional area is not different between centrally and non‐centrally nucleated fibres (Supporting information Figure S2).

In old muscle, SBB‐positive fibres have lower cross‐sectional area than SBB‐negative ones (Figure 2b). Moreover, myofibre diameter was significantly associated with frequencies of senescent nuclei as indicated by high levels of nuclear p21 (Figure 2c) or low HMGB1 (Figure 2d) such that fibres with more senescent nuclei had lower diameters. These results suggest the intriguing possibility that the presence of senescence‐like nuclei (as indicated by high p21 and loss of HMGB1) could well contribute to age‐associated muscle fibre thinning.

### 2.3 A xenotransplant model to study effects of senescent cells in vivo

**Figure 3:**
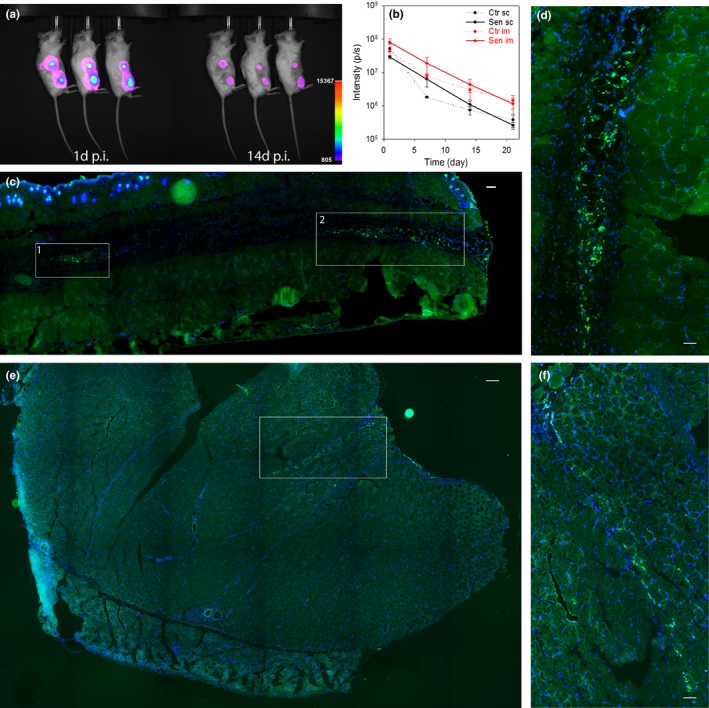
A xenotransplant model to study effects of senescent cells in vivo. (a) 7.5 × 10^4^ luciferase‐expressing senescent human fibroblasts were transplanted sc and im into one flank of NSG mice (aged 5 months) and were visualized after 24 hr (left) or 14 days (right) by chemoluminescence. (b) Chemoluminescence intensity from xenotransplanted senescent and young cells at sc and im sites at the indicated times after transplantation. Data are mean ± *SE* from five animals. (c) Representative fluorescence tilescan from a skin cryosection showing two depots of EGFP‐positive xenotransplanted senescent cells between dermis and subcutaneous tissue 3 weeks postinjection (boxed areas). Bar equals 100 μm. (d) Box 2 from c at higher magnification. Bar equals 30 μm. (e) Fluorescence tilescan from gastrocnemius muscle showing a single depot of EGFP‐positive xenotransplanted senescent cells (boxed area) 3 weeks postinjection. Bar equals 100 μm. (f) Boxed area from e at higher magnification. Bar equals 40 μm

Our aim was to study the effect of replicatively senescent cells onto senescence in the surrounding tissue. However, mouse cells immortalize spontaneously with frequencies as high as 10^−3^ (Espejel & Blasco, 2002), which might compromise an autologous senescent cell transplant mouse model. In contrast, the proliferation arrest in senescent human fibroblasts is very stable. Transplanting either radiation‐induced senescent mouse preadipocytes, autologuous senescent ear fibroblasts or radiation‐induced senescent human preadipocytes intraperitoneally (the latter into SCID‐beige mice) had very similar effects on physical dysfunction in the recipient mice (Xu et al., 2018). SASP chemokine/cytokine composition was very similar in replicatively senescent human fibroblasts and mouse ear fibroblasts rendered senescent by irradiation (Supporting information Figure S3A). Senescent fibroblasts that were injected intraperitoneally into normal or SCID mice induced a strong immune reaction mediated primarily by p16‐positive macrophages (Hall et al., 2016). Therefore, we decided to use the more severely immunodeficient NOD scid gamma (NSG) mice that are defective in macrophages amongst other immune responses and injected these with replicatively senescent MRC5 human fibroblasts. Transplanted cells were labelled with luciferase and EGFP (MRC5‐GFP+Luc+) and either used as controls at population doubling level below 30 or grown to replicative senescence. Replicative senescence was established by virtual absence of proliferative activity (<0.1 PD/week over 4 consecutive weeks), induction of a SASP (Supporting information Figure S3A) and >80% positivity for senescence‐associated β‐galactosidase (not shown). Chemiluminescence intensity was equal in senescent and non‐senescent cells and increased linearly with cell number (Supporting information Figure S3B). A total of 75,000 cells each were transplanted in parallel subcutaneously and intramusculary into the same flank of the animal, while the contralateral flank served as a no‐injection control. The luminescence signal decreased exponentially over time with a half‐life time of about 3 days (Figure 3a, b). There was no preferential clearance of senescent cells over the observation period of 3 weeks, suggesting ineffective engraftment as the major reason for loss of the transplanted cells.

To enhance exposure of the mouse tissues to signals from xenotransplanted cells, transplantations were repeated weekly for a total of three times. 3 weeks after the last transplantation, mice were culled and tissues were exhaustively sectioned. In muscle and skin, one section every 160 μm was subjected to in situ chemoluminescence imaging. Positive sections were then imaged for EGFP fluorescence. If EGFP‐positive cells were identified (Figure 3c–f), confirming the chemoluminescence result, adjacent sections were used for analysis of senescence markers in the mice tissues.

### 2.4 Transplanted senescent cells induce localized senescence of myofibres and dermal fibroblasts in vivo

**Figure 4:**
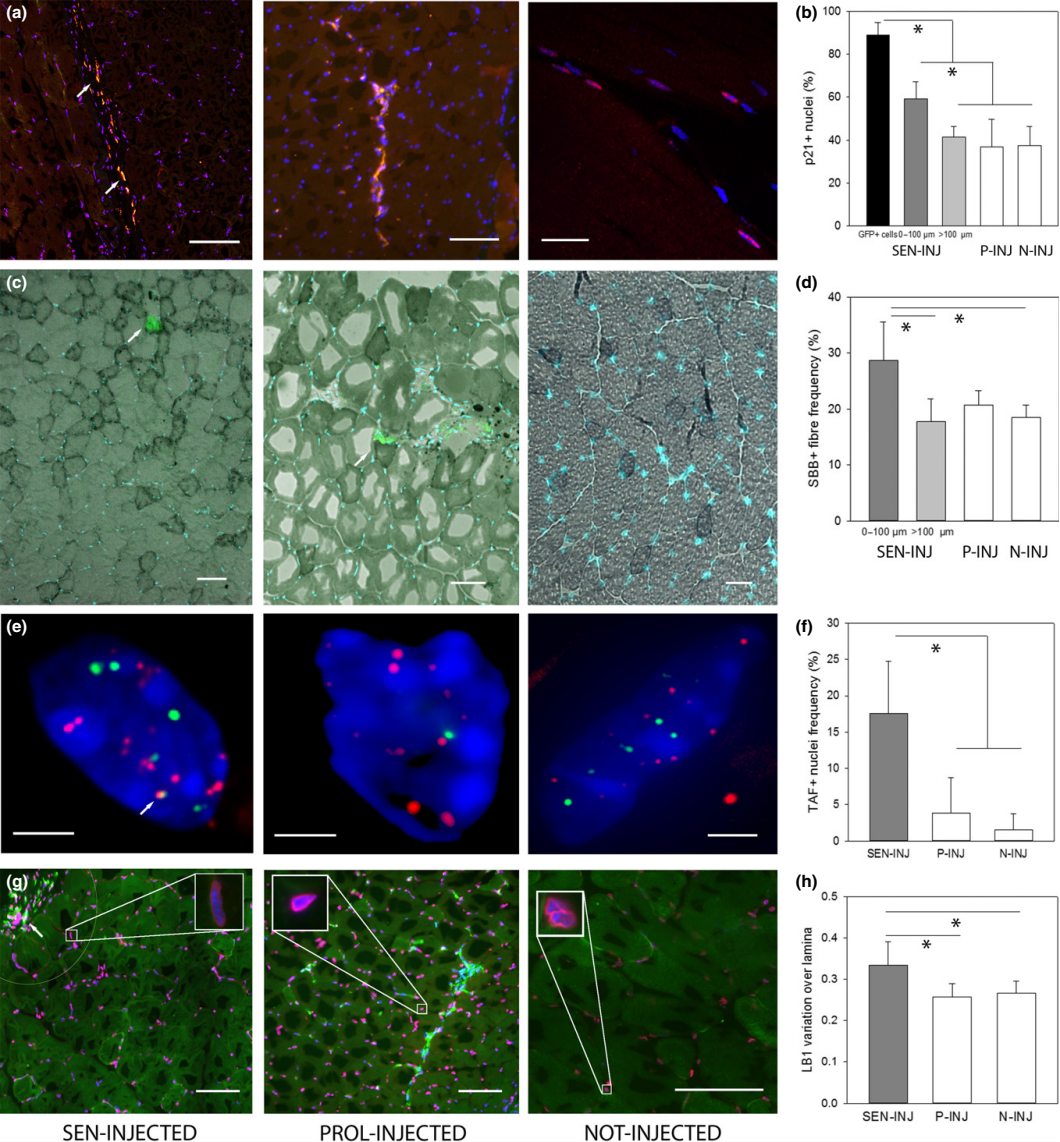
Transplanted senescent cells induce senescence in adjacent myofibres in vivo. (a) Representative p21 immunofluorescence images of muscles transplanted as indicated. Xenotransplanted fibroblasts appear yellow being both GFP (green)‐positive and p21(red)‐positive (arrows). Blue: DAPI. Scale bar 100 μm, except non‐inj: 30 μm. (b) Frequencies of p21‐positive nuclei. Xenotransplanted senescent cells (black bar), myocyte nuclei next to transplanted senescent cells (less than 100 μm away, dark grey) and more than 100 μm away (light grey) as well as myocyte nuclei close to proliferating xenotransplanted cells (P‐INJ, white bar) and from the non‐injected flank (N‐INJ, white bar). (c) Overlay of fluorescence images showing transplanted cells (green, arrow) and nuclei (blue, DAPI) with bright field images showing SBB‐positive myofibres (dark). Scale bar 50 μm. (d) Frequencies of SBB‐positive myofibres in relation to transplanted cells. Labels as in a. (e) TAF staining on myofibre nuclei in the vicinity (or not) to xenotransplanted cells. Red: telomeres, green: γH2AX. Arrow indicates a TAF. Bars equal 2 μm. (f) Frequencies of TAF‐positive myofibre nuclei in relation to xenotransplanted cells. (h) Representative LB1 immunofluorescence (red). Transplanted cells appear yellow (arrows). Blue: DAPI. The circle marks the area closer than 100 μm to the xenotransplanted cells. Insets highlight individual nuclei at higher magnification. Bars 75 μm. (i) Normalized pixel‐to‐pixel variation of laminar LB1 fluorescence intensity in myocyte nuclei in relation to xenotransplanted cells. All data are mean ± *SD* from five mice in the SEN‐INJ and P‐INJ groups and from two to 10 mice in the N‐INJ group. *p < 0.05 (one‐way ANOVA)

Having identified the xenotransplanted senescent fibroblasts, we next measured the senescence markers in adjacent myofibres. Almost all transplanted senescent fibroblasts were p21‐positive (Figure 4a). Frequencies of p21‐positive myofibre nuclei were highest adjacent to xenotransplanted senescent cells. They were equally low further away from transplanted senescent cells, in muscles injected with non‐senescent fibroblasts and in non‐transplanted contralateral muscle (Figure 4b). There were also more SBB‐positive fibres (Figure 4c, d) adjacent to transplanted senescent cells, while SBB‐positive fibre frequencies were lower in myofibres further away and in muscles transplanted with young cells or sham‐treated. TAF‐positive myofibre nuclei were much more frequent in muscle injected with senescent cells (Figure 4e, f). There was no significant effect on frequencies of lamin B1‐positive cells, but variation of the lamin B1 signal along the nuclear lamina was increased in senescence‐injected muscle (Figure 4g, h). There was no significant effect on Hmgb1 nuclear staining intensity (not shown). Muscles from NSG mice showed generally lower cytokine levels than wild‐type muscles (Supporting information Figure S1C). At 3 weeks after the last injection, cytokine levels were not different between senescent cell‐transplanted, young cell‐transplanted or untreated muscles (Supporting information Figure S1C). This was as expected given that we did not see induction of SASP at old age (Supporting information Figure S1D) and that numbers of senescent fibroblasts remaining in the tissue at 3 weeks after the last injection were as low as about 10^3^ cells (Figure 3b).

There were more myofibres with centrally located nuclei adjacent to xenotransplanted senescent cells than in non‐transplanted muscle (Supporting information Figure S4A). However, fibre cross‐sectional area was not significantly influenced by transplanted senescent cells (Supporting information Figure S4B).

Similar to muscle, subcutaneous injection of senescent MRC5‐GFP+Luc+ cells decreased frequencies of mouse dermal fibroblasts positive for the proliferation marker Ki67 in the vicinity of the injection site (Supporting information Figure S4C). Moreover, dermal fibroblasts were more often TAF‐positive (Supporting information Figure S4D) and p21‐positive (Supporting information Figure S4E) next to xenotransplanted senescent cells, while injection of non‐senescent MRC5 cells had no effect as compared to the contralateral flank (Supporting information Figure S4C–E).

### 2.5 A model for the quantitative assessment of senescent cell accumulation and clearance in vivo

During aging, senescent cells may be generated de novo, that is, by replicative exhaustion or cell‐autonomous stress, or via bystander signalling from pre‐existing senescent cells. This accumulation is partly counteracted by immune‐mediated turnover of senescent cells, resulting in a slow accumulation as net effect. In mouse liver, this accumulation is remarkably linear with age at least over the first half of lifespan (Jurk et al., 2014), suggesting that rates of accumulation and degradation may be approximated as constants over short time spans. A simple model for the net accumulation rate N is then:

(1) N=S+B−I

with *S*: spontaneous accumulation rate due to replicative exhaustion and damage, *B*: rate of senescent cell accumulation due to the bystander effect and I: rate of immunodegradation of senescent cells.

NSG mice do not have a functional immune defence. Therefore, we expect *I*_NSG_ = 0. Moreover, the bystander effect is dependent on signalling through SAMD‐generated ROS activating the SASP, primarily its pro‐inflammatory arm via induction of NF‐κB (Acosta et al., 2013; Coppe et al., 2008; Nelson, Kucheryavenko, Wordsworth & von Zglinicki, 2018). Both SASP and SAMD are effectively suppressed by inhibition of mTOR‐dependent nutrient signalling, for example, under dietary restriction (DR) or DR mimetics (Correia‐Melo et al., 2016; Ogrodnik et al., 2017). Accordingly, transcription of the vast majority of NF‐κB target genes including the NF‐κB subunits Nfkb1, Nfkb2 and Nfkbia is downregulated in mouse livers after short (3 months) dietary restriction (Supporting information Figure S5). Therefore, we expect for the bystander effect under DR *B*_DR_ ~ 0. Equation (1) then simplifies to

(2) $$N_{\mathrm{NSG},\mathrm{DR}}=S;N_{\mathrm{NSG},\mathrm{AL}}=S+B;\;N_{\mathrm{wt},\mathrm{DR}}=S-I;N_{\mathrm{wt},\mathrm{AL}}=S+B-I$$

**Figure 5:**
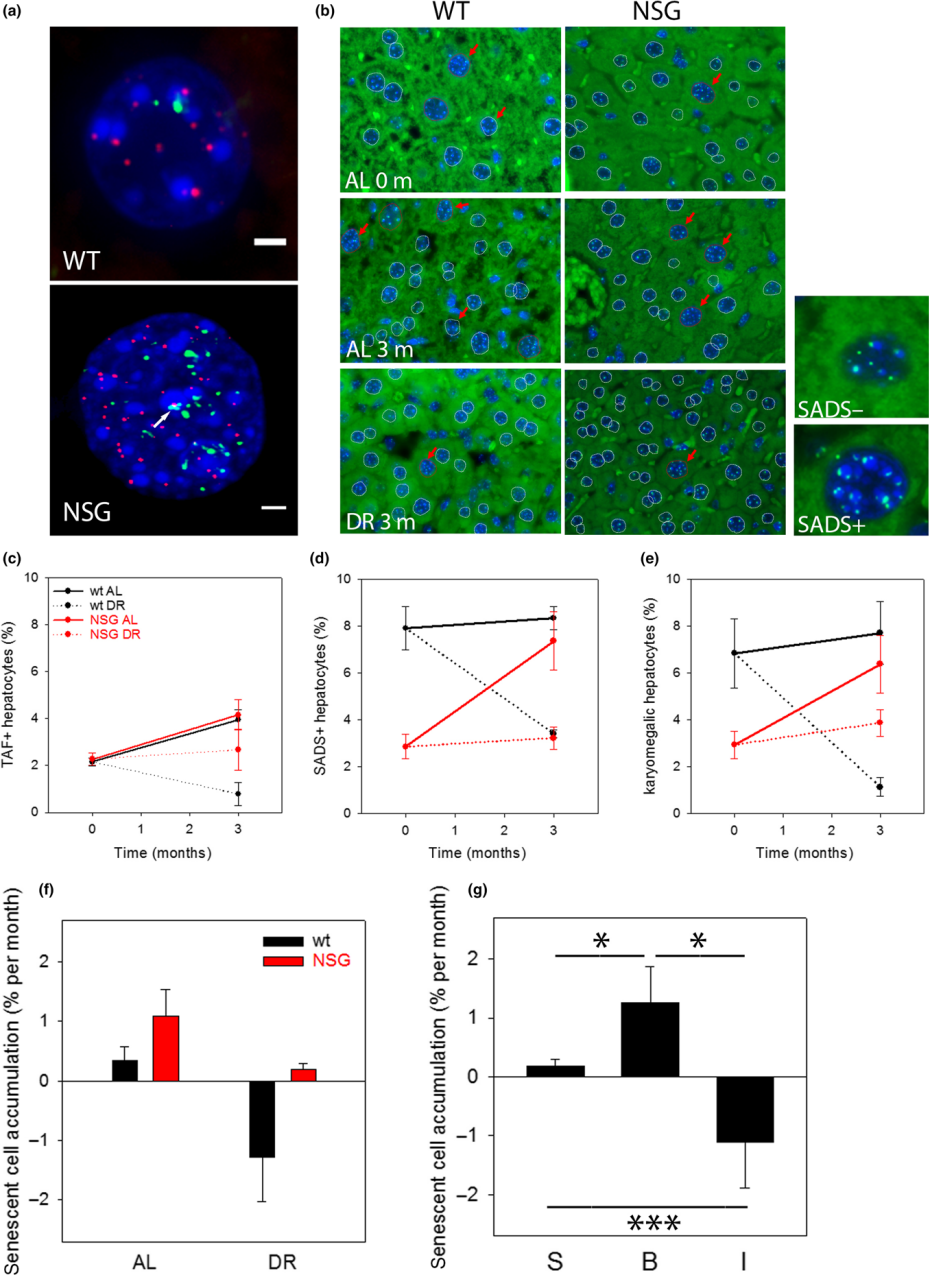
The bystander effect is a major driver of senescent cell accumulation with age in mouse liver. (a) Immuno‐FISH staining for telomeres (red) and γH2AX (green) in hepatocytes from wt and NSG livers. Signal co‐localization (TAF) is marked by an arrow. Scale bar equals 2 μm. (b) SADS and karyomegaly in liver from wt and NSG livers. Red arrows mark SADS‐positive hepatocytes (with four or more decondensation events, examples of SADS+ and SADS− nuclei at higher magnification on the right), outlines mark hepatocyte nuclei (red: karyomegalic, white: non‐karyomegalic). AL; ad libitum fed, DR: dietary restricted for 3 months. (c) Frequencies of TAF‐positive hepatocytes in wt (C57Bl6, black)) and NSG mice (red) at start of the experiment and after 3 months AL or DR feeding. (d) Frequencies of SADS‐positive hepatocytes. (e) Frequencies of hepatocytes showing karyomegaly. Data in (c) – (e) are mean ± *SE* from five mice per group. Differences between AL and DR at the end of the experiments are significant (*p* < 0.05, *t* test) for all markers except TAF in NSG. (f) Net senescent hepatocyte accumulation rates in wt and NSG mice. Data are averages ± *SD* from the three markers shown in (c) – (e). A two‐way ANOVA shows significant differences between wt and NSG (*p* = 0.003) and between AL and DR (*p* = 0.001) with no significant interaction between both factors. (g) Data given in (f) were used to calculate the impact of “spontaneous” senescence (S), the bystander effect (B) and immunosurveillance (I) on senescent hepatocyte accumulation rates. S, B and I are all different from each other (*p* < 0.05, one‐way ANOVA, Holm–Sidak post hoc test)

To test these expectations and to derive an estimate of the relative impact of spontaneous generation, bystander effect and immunodegradation on the net accumulation of senescent cells in a tissue, we performed short‐term (3 months) DR experiments (40% food reduction) in both wild‐type and NSG mice in parallel. NSG mice that have shorter lifespans accumulate senescent cells faster than wild‐type mice in agreement with previous data (Jurk et al., 2014). Therefore, DR was started at a younger age (3 months) in NSG mice than in wild‐types (12 months). We measured frequencies of senescent hepatocytes in livers using three markers: TAF (Hewitt et al., 2012; Jurk et al., 2014; Ogrodnik et al., 2017) (Figure 5a), senescence‐associated distension of satellites (SADS; Swanson, Manning, Zhang & Lawrence, 2013; Ogrodnik et al., 2017) and karyomegaly (Aravinthan & Alexander, 2016; Ogrodnik et al., 2017) (Figure 5b). There was good quantitative agreement between the three independent markers (Figure 5c–e). The lower TAF estimate for wild‐type mice is probably due to an over‐restrictive threshold setting (>2 TAF required to count a cells as senescent), as noted before (Jurk et al., 2014). Net accumulation rates *N*_wt,AL_, *N*_NSG,AL_, *N*_wt,DR_ and *N*_NSG,DR_ were calculated as averages from the three markers (Figure 5f). It follows from equation (2) that

$$S=N_{\mathrm{NSG},\mathrm{DR}}$$

$$B=N_{\mathrm{NSG},\mathrm{AL}}-N_{\mathrm{NSG},\mathrm{DR}}\;\text{or}\;B=N_{\mathrm{wt},\mathrm{AL}}-N_{\mathrm{wt},\mathrm{DR}}$$

$$I=N_{\mathrm{wt},\mathrm{DR}\;}-N_{\mathrm{NSG},\mathrm{DR}\;}\;\text{or}\;I=N_{\mathrm{wt},\mathrm{AL}}-N_{\mathrm{NSG},\mathrm{AL}}$$

Results (Figure 5g) indicate that the impact of the bystander effect on generation of senescent hepatocytes under the assumptions given above would be much greater than that of replication and other stresses. In livers of immunocompetent mice at the examined age, immunosurveillance (*I*) essentially balances the generation (*S* + *B*) of senescent cells.

## 3 DISCUSSION

We show here that in skeletal muscle from old mice, cellular markers of sarcopenia (low fibre cross‐sectional area, frequent centrally located nuclei) and cellular markers of a senescent cell phenotype are both enhanced and tend to correlate with each other. A senescent phenotype in old muscles was corroborated at tissue level by increased mRNA abundance of the major senescence marker p16 and multiple SASP proteins. Surprisingly, this was not translated into increased SASP protein abundance as measured by cytokine array in old muscles. Due to the low number of tissue samples and the fact that qPCR and cytokine arrays were performed on samples from different animals, we cannot exclude technical reasons for this discrepancy. Further in vitro work will be necessary to clarify whether low translation efficiency or fast release of SASP factors into the circulation contributes to this result. It is well established that muscular IL‐6 transcription and release can be stimulated by factors like exercise, high NO or low glycogen (Nielsen & Pedersen, 2008). In any case, the low abundance of pro‐inflammatory SASP factors in old muscle invites speculation about how senescent bystander effects in muscle are mediated. Senescent cells are known to release a host of other factors in addition to the “classical” SASP, including various proteins, oxidized lipids (Ni et al., 2016) and reactive oxygen species (ROS; Korolchuk et al., 2017; Passos et al., 2010). ROS has been implicated in mediating senescent bystander effects in vitro (Nelson et al., 2012, 2018). Attenuated redox regulation contributes significantly to age‐related sarcopenia (McArdle & Jackson, 2017). Experiments using SASP inhibitors and ROS scavengers might help to elucidate the relevant transducers of the bystander signal in muscle. However, this might be an extremely complex task, as all these signals are highly interconnected and kinetically interdependent (Hoare et al., 2016; Jurk et al., 2014; Korolchuk et al., 2017; Passos et al., 2010).

Our findings in aged skeletal muscle are in apparent contrast to previous results (Jeyapalan, Ferreira, Sedivy & Herbig, 2007), who found no increase of 53BP1‐positive DNA damage foci and no increase of TIF (telomere‐induced foci)‐positive nuclei in baboon skeletal muscle with age. However, Jeyapalan et al. defined TIF‐positive cells differently from us and, importantly, in our study five independent markers of senescence are similarly increased in muscles from old animals. Our results are reminiscent of the development of a senescent‐like phenotype during aging in another major postmitotic cell type, neurons. In both central and peripheral neurons from old mice, a telomere‐induced DNA damage response activates p21 that drives a multitude of changes associated with cell senescence (Jurk et al., 2012). Our present data suggest that neurons are no exception.

In vitro, senescent cells can induce senescence in bystander cells through a signalling network involving complex interactions between ROS and SASP factors, especially TGF‐β family ligands (Acosta et al., 2013; Nelson et al., 2018). That such a bystander effect may also be active in vivo has been indirectly inferred from senescent cell clustering in multiple tissues (Acosta et al., 2013; Nelson et al., 2018). Here, we show that senescent cells when transplanted into muscle or skin induced senescence in surrounding cells of the host tissues. This effect was specific to senescent cells, and transplanted proliferation‐competent fibroblasts did not enhance host tissue senescence. If there was transient induction of senescence during the healing of the injection wounds, it was too small to induce an increase that persisted for 3 or more weeks, that is, after completion of wound healing. Over the observation period of maximally 5 weeks, the bystander effect from senescent xenotransplanted cells was also localized: In contralateral tissues and even in the same tissue at some distance from the injection site, there was no significant enhancement of senescent cells. Furthermore, senescence marker increases following xenotransplantation were generally less than those seen in old animals. Specifically, myofibre cross‐sections as the most direct marker of possible sarcopenia were not significantly decreased. Similarly, we did not observe thinning of the epidermis close to sites of injection of senescent cells (not shown). Low engraftment efficiency and low numbers of the xenotransplanted cells together with a relatively short time between xenotransplantation and analysis are the most probable reasons for the paucity of functionally relevant effects. This is in agreement with Xu et al. (2018) who in their intraperitoneal transplantation model using senescent preadipocytes recorded significant physical effects only if transplanting at least 5 × 10^5^ senescent cells. Thus, while it is tempting to speculate that the senescence‐like myofibre phenotype might provide the causal signal for fibre degeneration and sarcopenia, more conclusive intervention experiments will be necessary to prove this speculation. However, even under these circumstances, the transplanted senescent cells induced significant bystander effects in both tested tissues.

A limitation of our study is that we tested the effects of transplanted senescent cells in males only. However, a recent study (Xu et al., 2018) confirmed the existence of an in vivo bystander effect also in females.

To estimate the relative importance of the bystander effect for accumulation of senescent cells in vivo, we compared frequencies of senescent hepatocytes in livers from immunocompromised and immunocompetent mice before and after a relatively short period of dietary restriction, which effectively suppresses the signalling pathways that induce the bystander effect (Blagosklonny, 2010; Correia‐Melo et al., 2016; Ogrodnik et al., 2017; Wang et al., 2010). This showed that already in livers from relatively young mice, the bystander effect contributed strongly to senescent cell accumulation.

Evidently, our model is limited by the simplicity of its underlying assumptions. In the long run, none of the senescent cell accumulation rates will remain constant. Rather, both bystander effect rate B (due to increasing numbers of senescent inducer cells) and cell‐autonomous rate S (due to decreasing DNA repair efficiency and increasing replicative exhaustion) are expected to increase with age, while immunosenescence might decrease the capacity for immunosurveillance. However, our previous results showed linear senescent cell net accumulation in mouse liver with age (Jurk et al., 2014), justifying the assumption of constant rates over the short observation period of 3 months used here. The relative impact of B vs S might, however, be different at old age. It seems probable that with increasing numbers of prevalent senescent cells, the bystander contribution will further increase.

Another simplifying assumption in our model is that the cell‐autonomous rate of senescent cell generation S was set as equal under all four conditions. NSG mice are deficient in the DNA repair enzyme DNA‐PK, leading to increased accumulation of DNA damage and possibly increased S. Dietary restriction, as well as dietary restriction mimetics like rapamycin or metformin, might suppress not only NF‐κB‐driven bystander signalling but also induction of cell‐autonomous DNA damage, because these interventions are well known to reduce ROS levels (Correia‐Melo et al., 2016; Lesniewski et al., 2017; Park & Shin, 2017). However, DR‐mediated reduction of ROS is at least partially a secondary consequence rather than simply a direct cause of reducing net senescent cell accumulation (Correia‐Melo et al., 2016; Passos et al., 2010). A further potential limitation of our model stems from the fact that NSG mice retain some rudimentary immune function, thus I_NSG_ might not be completely zero. Unfortunately, variation in the experimental data is too large to enable a meaningful evaluation of more complex models. Therefore, our calculations should be regarded as rough approximations only.

Clearly, replicative senescence might play a larger role in highly proliferating tissues like spleen, gut epithelium or testis. However, it seems safe to assume that even there the bystander effect will make a significant contribution to senescent cell accumulation with age.

Finally, our data make an interesting contribution to the ongoing discussion about “senolytic” versus “senostatic” drugs. It has been shown that senolytic interventions, that is, treatments that selectively kill senescent cells, alleviate a wide range of age‐associated diseases and disabilities (Baker et al., 2016; Ogrodnik et al., 2017; Xu et al., 2017). In some cases, lasting effects have been seen after short treatment periods, and it has been argued that even a short senolytic treatment by killing sufficient numbers of senescent cells might be sufficient to “reset the clock.” This is important as short treatment periods enable better acceptance of toxic side effects. On the contrary, “senostatic” drugs including the DR mimetics rapamycin and metformin suppress the senescent phenotype in a reversible manner (Correia‐Melo et al., 2016). Senostatics do not induce senescent cell apoptosis. In fact, hepatocyte apoptosis frequencies as measured by cleaved caspase 3 assay following dietary restriction for 3 months are below 10^−3^ and not enhanced over those in ad libitum fed mice (data not shown). Therefore, it is generally assumed that senostatics would need to be given permanently to be effective. However, our data show that an intervention that is senostatic in vitro actually may reduce senescent cells frequencies in vivo in an immunocompetent host almost as effective as a senolytic drug due to suppression of the bystander effect under persistence of immunosurveillance. If reduction of senescent cell frequencies is important for the beneficial effects of DR and DR mimetics on healthspan, our results would predict that “senostatic” interventions over relatively short terms might show significant benefits. In fact, lasting beneficial effects from short‐term DR have been frequently documented (Cameron, Miwa, Walker & von Zglinicki, 2012; Ogrodnik et al., 2017; Selman & Hempenstall, 2012). It would be interesting to assess long‐term effects of short‐term treatments with DR mimetics in vivo.

## 4 EXPERIMENTAL PROCEDURES

### 4.1 Mice

All animal experiments were carried out in compliance with the Home Office regulations and FELASA guidelines. Wild‐type C57Bl6 mice were group housed. 40% dietary restriction was administered as described (Cameron et al., 2012). NSG mice were purchased from Charles River and housed under 12 hr day/night cycle, in individually ventilated cages. We injected adult, 4‐ to 6‐month‐old, NSG male mice (*n* = 5) with 50 μl of 1.5 × 10^6^ cell/ml suspension of senescent MRC5‐GFP+Luc+ cells subcutaneously in the right flank and intramuscular in the right hindlimb. Control mice (n = 5) were injected with the same amount of proliferation‐competent MRC5‐GFP+Luc+ cells. Procedures were performed under general inhalational anaesthesia followed by administration of analgesic to provide pain relief after the intra‐muscular injection.

To verify localization of injected cells, mice were subjected to in vivo imaging using an IVIS Spectrum system. Injections were repeated two more times in weekly intervals, and cells were followed by weekly in vivo imaging for a total of 5 weeks after the first injection. Then, the site of injected cells was marked, mice were euthanized and skin and muscle tissues from injected and non‐injected sites were collected.

### 4.2 Cell culture

MRC5 primary human fibroblasts were cultured at 5% CO2, in ambient oxygen in DMEM supplemented with 10% foetal calf serum, 100 U/ml penicillin, 100 mg/ml streptomycin and 2 mM l‐Glutamine medium. To generate MRC5‐GFP+Luc +, early passage cells were transduced with pSLIEW (Bomken et al., 2011), a lentiviral vector expressing enhanced green fluorescent protein (EGFP) and luciferase (Luc). To evaluate efficiency of cells to luminesce, in vitro luciferase assays were performed on cells in a range of dilutions (150 to 2 × 10^4^ cells/well) in 96‐well plate incubated with 100 mM d‐Luciferin using a luminescent plate reader (BMG Labtech). To generate replicatively senescent cells, MRC5‐GFP+Luc + cells were cultured until cells reached a population doubling rate below 0.1 per week.

### 4.3 Tissue sectioning

Gastrocnemius and biceps femoris muscles and skin of both injected and non‐injected flanks were flash‐frozen in isopentane cooled with liquid nitrogen. Tissues were sectioned into 10 μm sections in a Leica CM1950 cryostat at −18°C to −20°C. Injected tissues were exhaustively sectioned, following a repeating pattern of collecting every second section. Slides were stored at −70°C until analysis.

### 4.4 In situ luciferase assay

Freshly sectioned slides were placed in a FujiLAS4000 chemiluminescent chamber (GE Healthcare Life Sciences). One section was analysed for each 160 μm distance. After the slides were placed in the chamber, 10–20 μl (depending on section size) of 10 μM ATP and 100 mM d‐Luciferin solution in 1X‐PBS were applied and the luminescent signal was collected for 3 min. Immediately after, tissue sections were fixed for 10 min in 4% paraformaldehyde (PFA) in PBS, washed and mounted with DAPI for screening for xenotransplanted EGFP‐positive cells.

### 4.5 Immunofluorescence and immuno‐FISH

Sections were thawed at room temperature for 5 min, fixed in 4% PFA in PBS for 10 min, washed 2 times for 5 min with 1X‐TBS and permeabilized with 0.1% Triton X‐100 in PBS for 10 min. To block non‐specific staining, tissues were incubated with 1% BSA and 1% gelatin in TBS at room temperature for at least 1 hr. Sections were incubated overnight at 4°C with primary antibody: anti‐lamin B1 (rabbit; ab16048; 1:1000 dilution), anti‐Hmgb1 (rabbit; ab18256; 1:500 dilution) or anti‐p21 (rabbit; ab7960; 1:250 dilution). Sections were washed three times for 5 min with 1X‐TBS and incubated with an anti‐rabbit IgG, Alexa Fluor^®^ 594 conjugated secondary polyclonal antibody (goat; A‐11037; 1:1000 dilution) for 1 hr at room temperature. Sections were mounted with Prolong Diamond mounting media with DAPI. Immuno‐FISH for staining of telomere‐associated DNA damage foci was performed as described (Hewitt et al., 2012).

### 4.6 Sudan Black B staining

Sudan Black B (SBB) staining was performed as described (Georgakopoulou et al., 2013), with minor modifications. In short, SBB powder was dissolved in 100% in ethylene glycol (7 mg/mL), covered with parafilm, in order to avoid evaporation, and stirred overnight. After, the solution was filtered through filter paper and through a Polyethersulfone Syringe Filter (30 mm membrane diameter, 0.22 μm pore size) and stored in an airtight container.

Cryosections were thawed at room temperature for 5 min, fixed in 1% PFA in PBS for 5 min, washed three times for 1 min with deionized water and then incubated in 100% ethylene glycol for 5 min. After that, two/three drops of SBB solution were dropped on a clean glass slide and, in order to avoid SBB precipitation, the slide containing the cryosections was placed, facing down, on the glass slide, with the cryosections in direct contact with the drops of SBB solution and left facing down for 3 hr at room temperature. The coverslip was carefully lifted, and excess SBB staining was removed with 2–3 quick rinses with deionized water. Tissues were then incubated for 3 min in 80% ethylene glycol, followed by several quick rinses with deionized water. Cryosections were counterstained with Nuclear Fast Red and mounted in Prolong Gold mounting media.

### 4.7 Image acquisition

For immunofluorescence, images were acquired using a Zeiss Axio Observer microscope equipped with a Yokogawa CSUX1 spinning disc head and a QuantEM camera; EC plan Neofluar (40×; Numerical Aperture (N.A.) 1.3) and Plan Apochromat (63×; N.A. 1.4) objectives were used. Z stacking was performed following Nyquist criteria.

For SBB‐ and H&E‐stained sections, images were acquired using a Nikon E800 wide field upright microscope equipped with a Leica DFC450C camera; 10× (N.A. 0.3) and 20× (N.A. 0.5) objectives were used. 8–12 fields were taken per tissue.

For immuno‐FISH‐stained sections, images were acquired using a DMi8 fluorescence inverted microscope with a 100× (N.A. 1.44) objective. Nyquist criteria z stacks were captured using a Hamamatsu Flash4 sCMOS camera. A minimum of 50 nuclei were imaged per tissue. Image acquisition was performed using LASX software (Leica) and deconvolved with Huygens software (SVI).

To assess senescence marker in relation to injection sites, tiling scans were generated using the tiling function of a DMi8 equipped with a motorized stage, collecting high‐resolution images over large areas to enable identification of injection sites and analysis of close and distant bystander cells.

### 4.8 Image analysis

Images were composed and edited with ImageJ software (https://imagej.nih.gov/ij/), optimal brightness and contrast adjustments were applied to the whole image; parameters for image analysis were quantified using the same software.

To assess myofibre cross‐section area, individual fibres were manually outlined and their cross‐sectional area (CSA) was measured. To quantify centrally nucleated (CNF) and SBB‐positive fibres in muscle sections, numbers of positive fibres per field were counted in 8–14 fields per animal.

For quantification of HMGB1‐ and p21‐positive nuclei, first a nuclear mask was created using the DAPI channel of the acquired image. Touching nuclei were separated, and mean nuclear fluorescence intensity was measured over the mask area. Nuclei were classified as positive, if their fluorescence exceeded a threshold defined as cytoplasmic fluorescence intensity + 2× (standard deviation of cytoplasmic fluorescence intensity) per image.

For lamin B1 analysis, a mask of the nuclear periphery was created from the DAPI channel using the Image Calculator function in ImageJ and inverted. This mask was used to measure the pixel standard deviation over the nuclear lamina and the mean fluorescence intensity in the lamin B1 channel. *SD* values were normalized to the mean intensity per nucleus.

To quantify γH2A.X‐positive nuclei, nuclei with distinct γH2A.X foci were manually counted. Myofibre or dermal nuclei were considered TAF‐positive when containing at least one telomere co‐localizing with a distinct γH2A.X focus, while at least two TAF were required for positivity in hepatocytes.

To measure the association between Hmgb1‐ or p21‐positive nuclei and fibre thickness, sections were co‐stained with WGA‐Al647 to aid the association of individual nuclei to the correct fibre. Moreover, oblique sections were chosen to see multiple nuclei per fibre, and minimum Feret diameter rather than cross‐sectional area was used to estimate fibre thickness. On average, 2‐3 nuclei per fibre were scored.

### 4.9 Cytokine arrays

Frozen muscle samples were pulverized on dry ice and homogenized in RIPA buffer with protease inhibitor cocktail (Roche, #11 836 153 001). Protein concentrations were adjusted to 1 mg/ml. Analysis was performed using the Mouse Cytokine/Chemokine 31plex Array MD31 (Eve Technologies).

For measurement of in vitro cytokine release, mouse ear fibroblasts (MAFs) were prepared and analysed as described (Jurk et al., 2014). Human MRC5 fibroblasts were grown to senescence as described above. Media was changed to 4 ml serum‐free media and collected after 2 days. Supernatant was centrifuged for 10 min at 400 *g* at 4°C and stored at −80°C for analysis using the Human Cytokine/Chemokine 65‐plex Panel (Eve Technologies).

### 4.10 qPCR

Frozen muscles were weighed and ground in liquid nitrogen. RNA was isolated from 5 to 15 mg of sample according to the Qiagen lipid RNA isolation kit protocol. Reverse transcription was performed using Superscript III Reverse Transcriptase (Invitrogen). qPCR was performed using primers as described (Baker et al., 2016; Ishaq, Schroder, Edwards, von Zglinicki & Saretzki, 2018) and SyBr Green (SensiFAST SYBR Hi‐ROX, Bioline). 18S was used as housekeeping gene. Annealing temperatures for all primers were 60°C. Expression is shown as 2^−ΔΔCt^ values.

### 4.11 DeepSeq and senescence biomarker analysis in liver

Gene expression and senescence biomarker analysis in liver was performed as described (Ogrodnik et al., 2017). NF‐κB target gene identities were taken from http://bioinfo.lifl.fr/NF-KB/. Expression data were normalized to the average expression level for each gene.

### 4.12 Statistics

For the assessment of a bystander effect, mice were given number codes and experiments in injected tissues were evaluated blindly, without knowing whether the injected cells were proliferative or senescent. All experiments were repeated at least with three biological replicates. Unless stated otherwise, all data were normally distributed and presented in bar graphs as mean ± standard deviation (*SD*). SigmaPlot 12.5 software (©Systat Software Inc.) was used for all statistical analyses. Two groups were compared by Student's *t* test (for normally distributed data) or by Mann–Whitney *U* test in cases of non‐parametric distribution. One‐way ANOVA followed by Holm–Sidak multiple comparisons was used to compare the expression of biomarkers around sites of injection. Cross‐sectional areas of CNFs and non‐CNFs in adult and old tissues were compared by two‐way ANOVA followed by Holm–Sidak multiple comparisons. Statistical significance was considered for P values below 0.05. Clustering and heatmap visualization was performed in PERSEUS (Tyanova et al., 2016).

## CONFLICT OF INTEREST

The authors have no conflict of interests to declare.

## AUTHOR CONTRIBUTIONS

OK generated the animal model. PdS, OK, MO, JG, AI, SM, GS and GN produced the data. SNG generated DeepSeq data. KC and SM performed the dietary restriction experiments. GN and TvZ evaluated the results and wrote the manuscript with input from all other authors. TvZ designed the study and directed the research.
