# Supplementary material for: The bystander effect contributes to the accumulation of senescent cells in vivo
Source: Aging Cell. 2018 Nov 21;18(1):e12848. doi: 10.1111/acel.12848 (PMC6351849; doi:10.1111/acel.12848)
Supplement: Supplementary file 1 [file ACEL-18-e12848-s001.docx]

**Supplementary Figures and Legends**

**
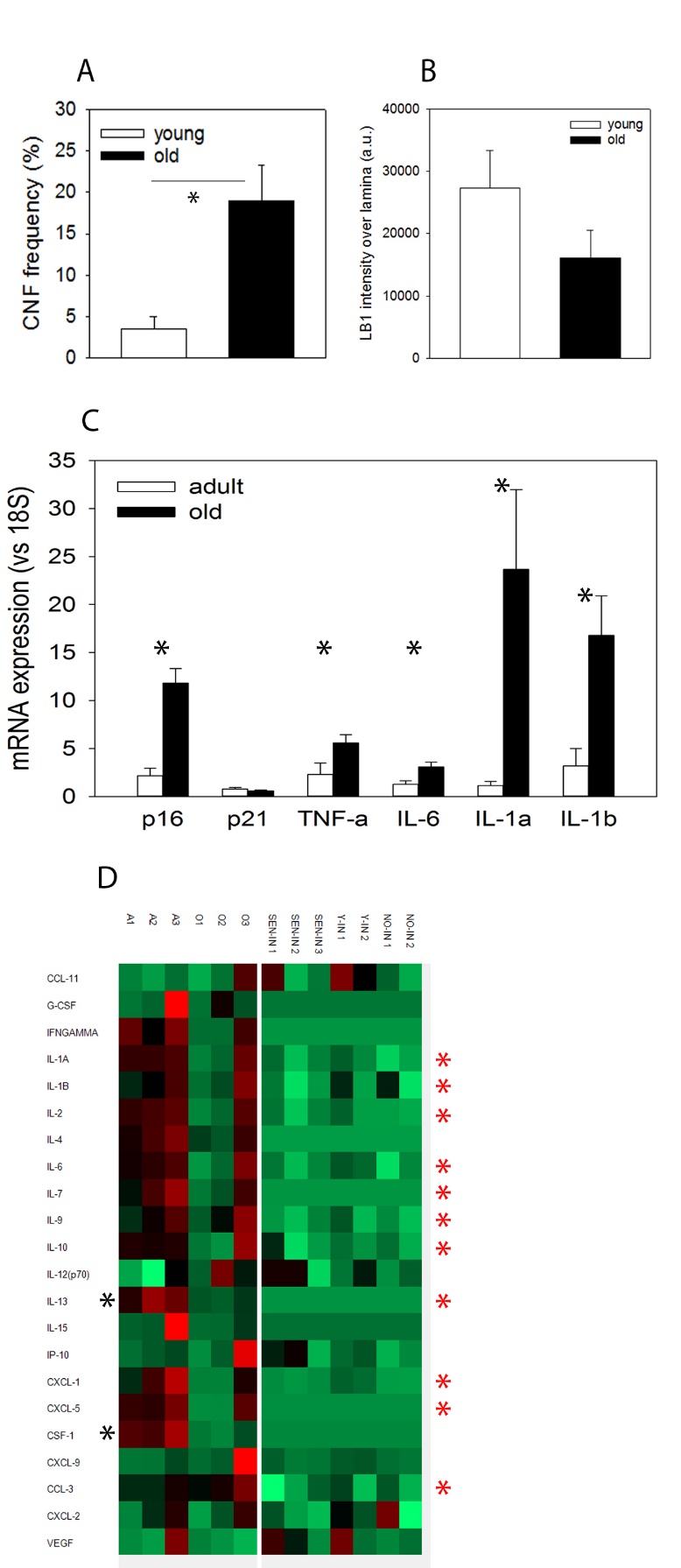
**

expression 2^-ΔΔCt^

**Suppl. Fig. S1: Further analysis of myofibre ageing and senescence.** A) Frequencies of centrally nucleated fibres in gastrocnemius from adult (8 months) and old (32 months) mice. B) Lamin B1 staining intensity over the nuclear lamina. C) mRNA levels of indicated senescence markers in adult and old muscle as measured by RT-qPCR with 18s RNA as standard. Data in A) to C) are M ± SE, n=5 (A, B) or n=4 (C). * p<0.05, t test. D) Levels of inflammation-related SASP factors as measured by cytokine array in adult (A) and old (O) muscles of C57Bl6 mice (left) and in NSG mice muscles (right) at 3 weeks after last injection with either senescent (SEN-IN) or proliferation-competent (Y-IN) fibroblasts or in the non-injected flank (NO-IN) of Y-IN mice. For comparison, data for each cytokine were z-scored over all 13 samples. Scale is from -1.5 (green) to +2 (red). Black stars indicate significant differences between adult and old and red stars indicate differences between C57Bl6 and NSG (*p<0.05, t test). There were no significant differences between the three NSG groups (ANOVA).

**
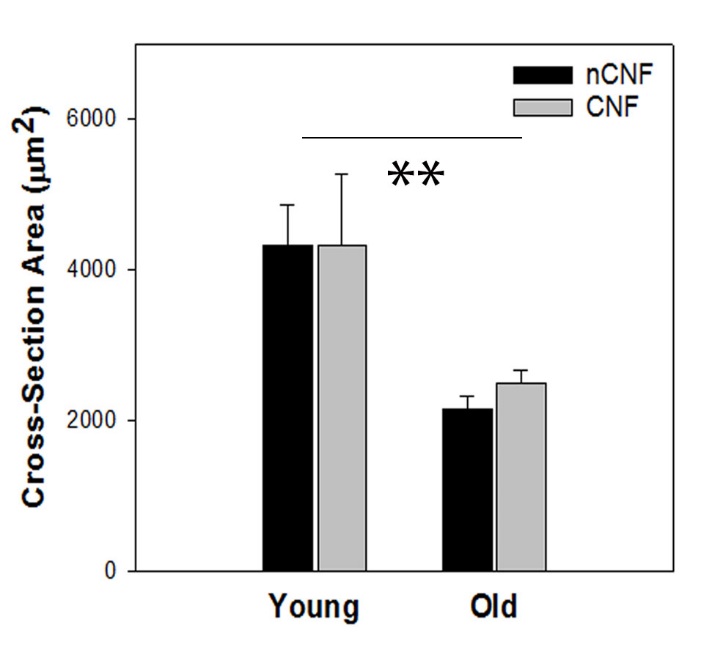
**

**adult old**

**Suppl. Fig. S2:** Average cross-sectional areas of centrally and non-centrally nucleated fibres. P<0.01 between adult and old, 2-way ANOVA.

A B

**
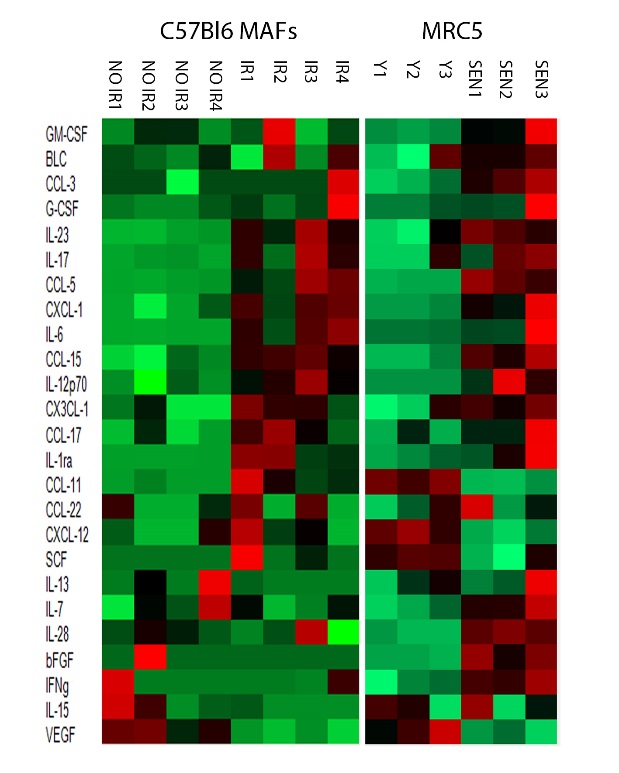

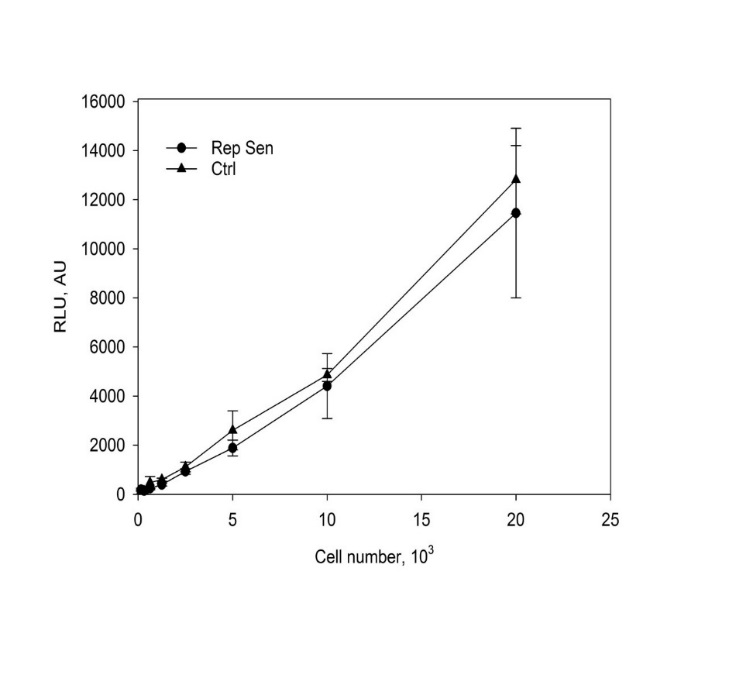
**

**Suppl. Fig. S3: Further characterisation of the xenotransplant model. A)** Cytokine/chemokine release from control (NO IR) and senescent (IR, two weeks after 10Gy IR) mouse ear fibroblasts (MAFs, left) and from control (Y) and replicatively senescent (SEN) human MRC5 fibroblasts (right). Concentrations were z-scored for each species. Scale is from -1.5 (green) to +2 (red). **B)** Equal chemiluminescence intensity (RLU) in senescent (Rep Sen) and non-senescent (Ctrl) human fibroblasts. Proliferating and replicatively senescent fibroblasts were trypsinised, counted and serially diluted in triplicate into a 96 well microplate. 1 mM (final concentration) d-luciferin was added to each well and luminescence measurements recorded. No significant difference in chemiluminescence was observed between control and Rep Sen cells at all cell concentrations.

**
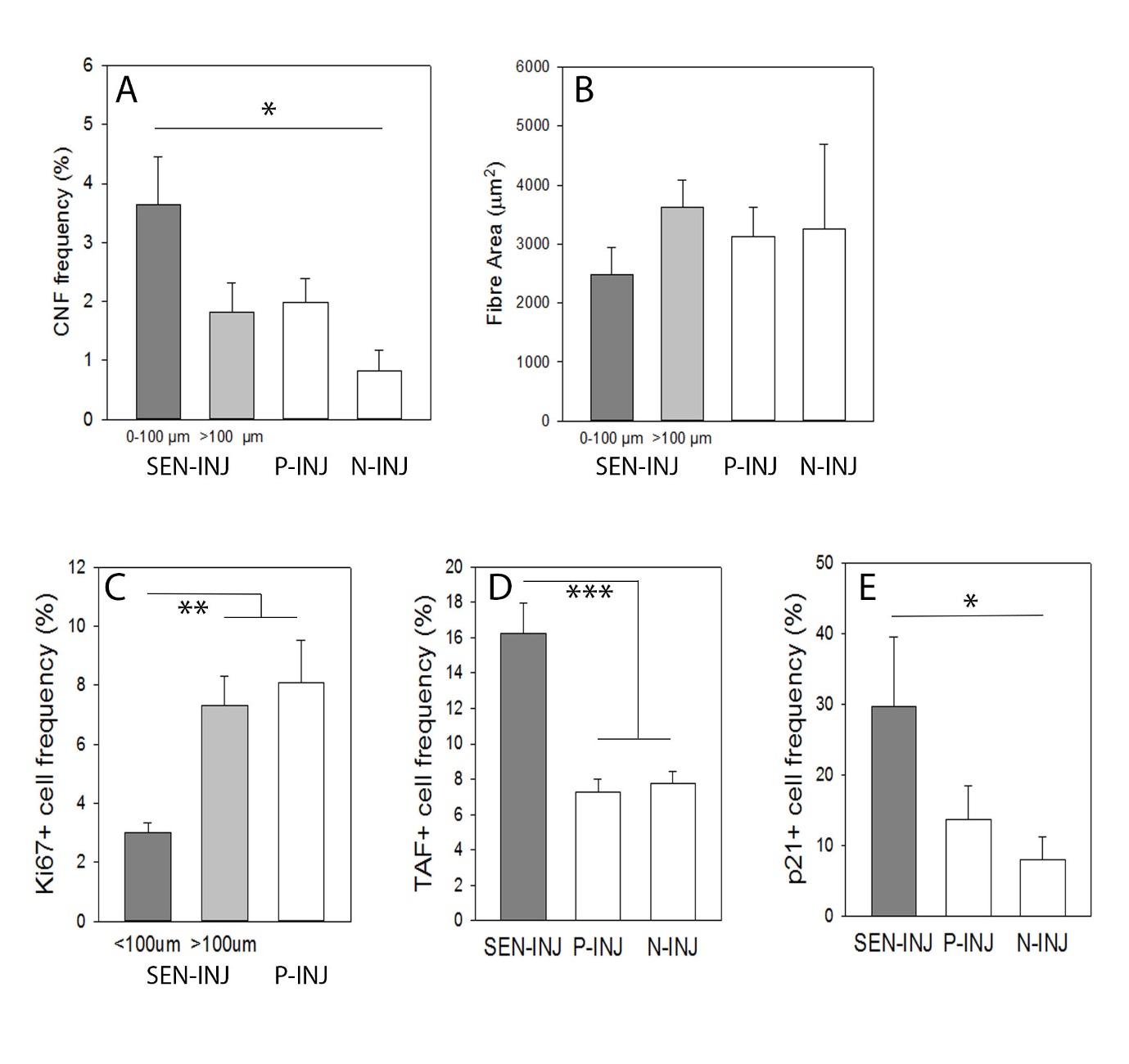
Suppl. Fig. S4: Further markers of the bystander effect in vivo.** A) Frequencies of centrally nucleated myofibres (CNF) in relation to xenotransplanted fibroblasts. Labels as in Fig. 3A. B) Myofibre cross-sectional area in relation to xenotransplanted fibroblasts. Labels as in Fig. 3A. C) Frequencies of Ki67-positive dermal mouse fibroblasts in relation to subcutaneously xenotransplanted senescent or non-senescent human fibroblasts. D) Frequencies of TAF-positive dermal mouse fibroblasts. E) Frequencies of p21-positive dermal mouse fibroblasts. Data are mean ± SE from 3 animals per group (6 animals for the N-INJ group).

**
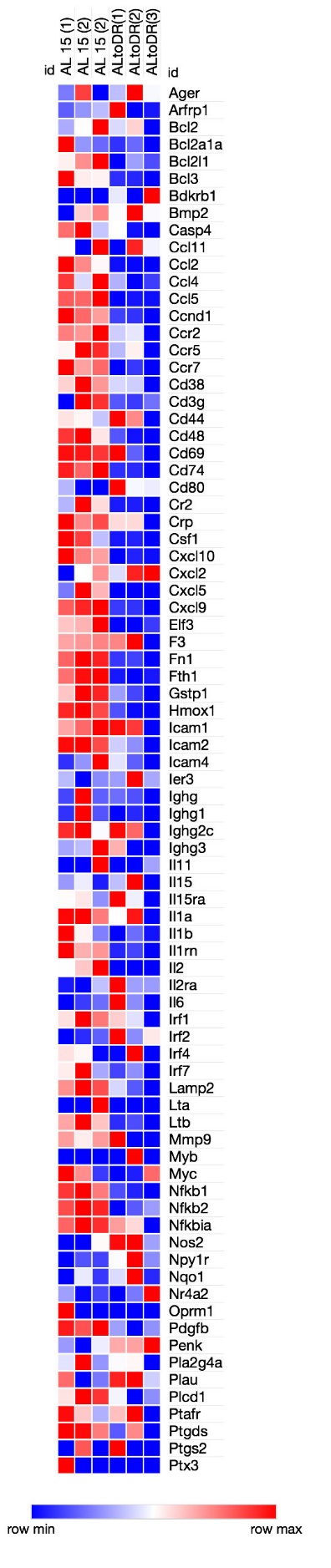
**

**Suppl. Fig. S5: Heatmap of NF-kB target gene expression in mouse livers**. DeepSeq was performed on liver tissue from 15 months old C57Bl6 mice fed ad libitum (AL15) or following 3 months dietary restriction (60% of ad libitum feeding, ALtoDR).
